# Supplementary material for: Role of NS1 antibodies in the pathogenesis of acute secondary dengue infection
Source: Nat Commun. 2018 Dec 7;9:5242. doi: 10.1038/s41467-018-07667-z (PMC6286345; doi:10.1038/s41467-018-07667-z)
Supplement: Supplementary file 1 — Supplementary Information [file 41467_2018_7667_MOESM1_ESM.pdf]

Role of NS1 antibodies in the pathogenesis of acute secondary dengue infection

Supplementary Information

Jayathileka et al

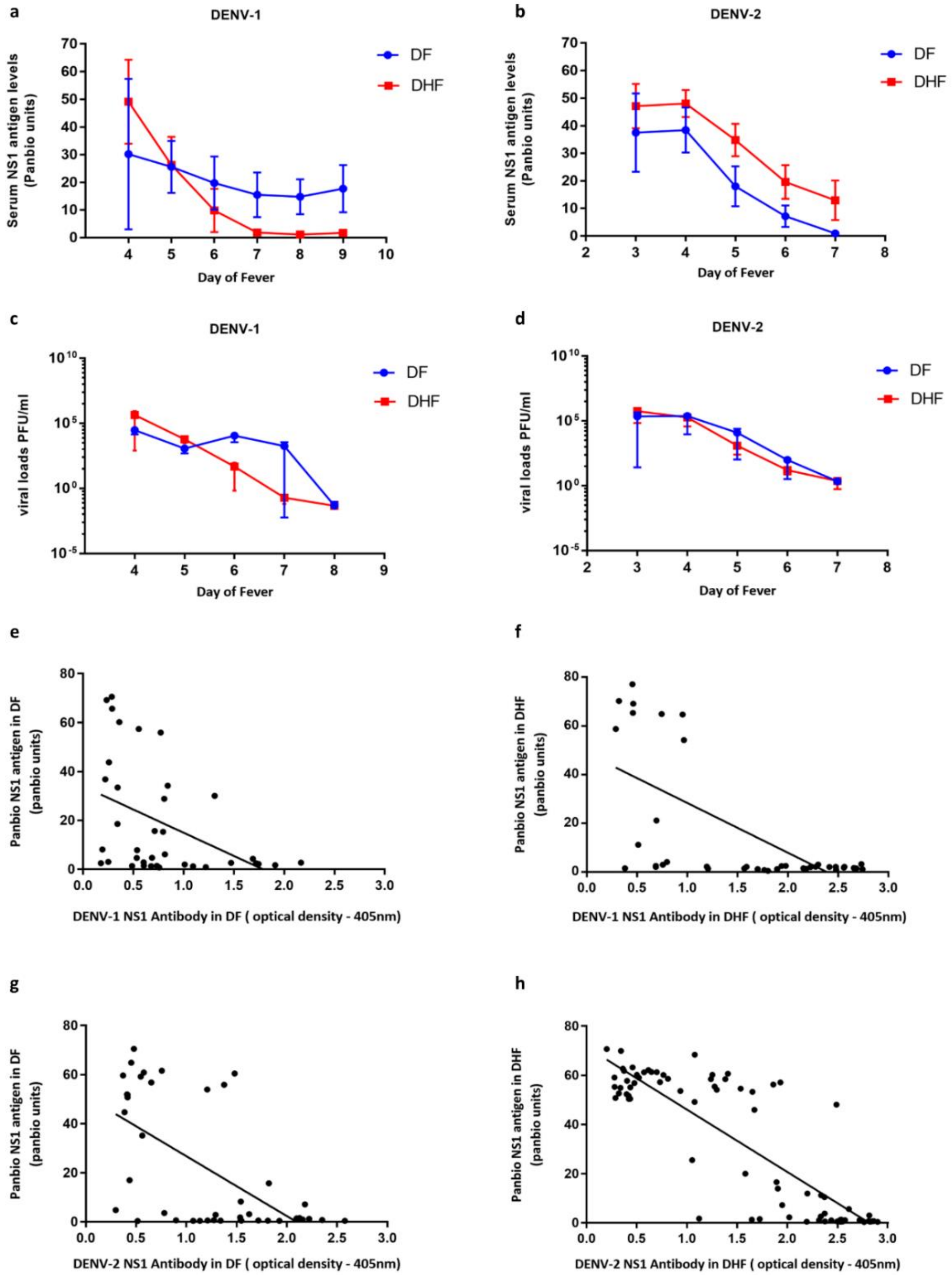
a
DENV-1
Serum NS1 antigen levels (Panbio units)
Day of Fever
DF
DHF
b
DENV-2
Serum NS1 antigen levels (Panbio units)
Day of Fever
DF
DHF
c
DENV-1
viral loads PFU/ml
Day of Fever
DF
DHF
d
DENV-2
viral loads PFU/ml
Day of Fever
DF
DHF
e
Panbio NS1 antigen in DF (panbio units)
DENV-1 NS1 Antibody in DF (optical density - 405nm)
f
Panbio NS1 antigen in DHF (panbio units)
DENV-1 NS1 Antibody in DHF (optical density - 405nm)
g
Panbio NS1 antigen in DF (panbio units)
DENV-2 NS1 Antibody in DF (optical density - 405nm)
h
Panbio NS1 antigen in DHF (panbio units)
DENV-2 NS1 Antibody in DHF (optical density - 405nm)

Supplementary Figure 1: Kinetics of NS1 antigen and viral loads correlation with NS1 antibodies (a) DENV1 NS1 antigen levels, measured in patients with an acute secondary DENV1 infection causing DF (n=10) or DHF (n=10) using ELISA. (b) DENV2 NS1 antigen levels were measured in patients with an acute secondary DENV2 infection causing DF (n=12) or DHF (n=23) using ELISA. Viral copy numbers were measured in patients with an acute secondary DENV1 (c) and DENV2 (d). Differences between serial values of NS1 antigen levels and viral loads in patients with DHF and DF were compared using the Holm-Sidak method. NS1 antigen levels and viral loads in patients with DHF are indicated in red, and in those with DF in blue. Error bars indicate mean and standard error of mean (SEM). Serum anti-NS1 antibody titres were measured throughout the course of illness by ELISA and correlated with serum NS1 antigen levels in patients with (e) DF due to DENV1, n= 10 (Spearmans' r=-0.45, p=0.005), (f) DHF due to DENV1, n=10 (Spearman's r=-0.60, p<0.0001), (g) DF due to DENV2, n=12 (Spearman's r=-0.50, p=0.0006), (h) DHF due to DENV2, n=23 (Spearman's r=-0.80, p<0.0001).

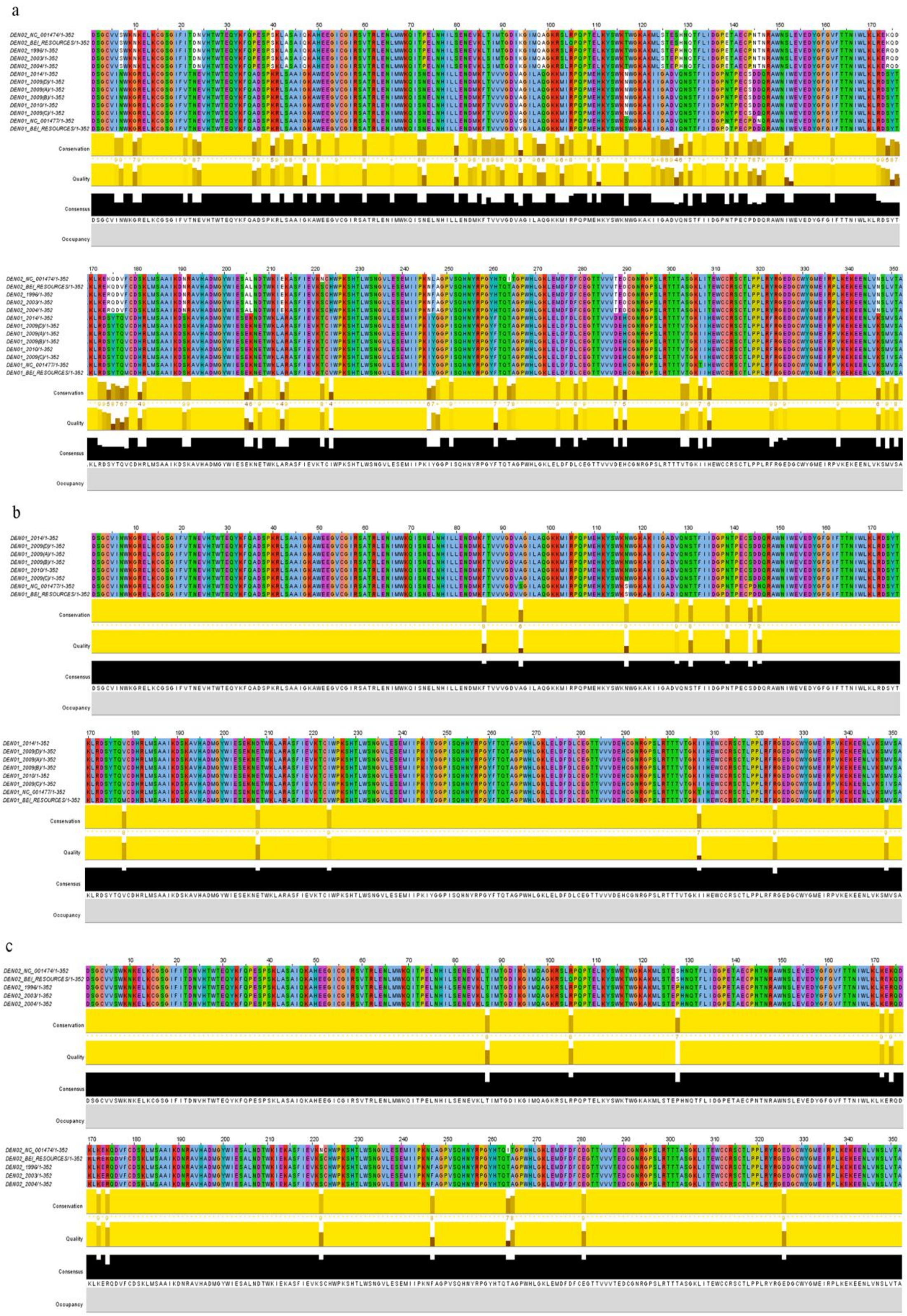
a
b
c
DEN02_NC_001474/1-352
DEN02_BEI_RESOURCES/1-352
DEN02_1996/1-352
DEN02_2003/1-352
DEN02_2004/1-352
DEN01_2014/1-352
DEN01_2009(D)/1-352
DEN01_2009(A)/1-352
DEN01_2009(B)/1-352
DEN01_2010/1-352
DEN01_2009(C)/1-352
DEN01_NC_001477/1-352
DEN01_BEI_RESOURCES/1-352
Conservation
Quality
Consensus
Occupancy

Supplementary Figure 2: Multiple alignment of DENV1 and DENV2 NS1 sequences. DENV1 and DENV2 sequences of identified in recent years in Sri Lanka were aligned including those which were used for mapping NS1 antigen epitopes using Clustal Omega (a), DENV1 sequences identified in recent years were aligned with the DENV1 sequence of NS1 used in the antibody epitope analysis (b) DENV2 sequences identified in in 2002-2008 were aligned with the DENV2 sequence of NS1 used in the antibody epitope analysis (c)

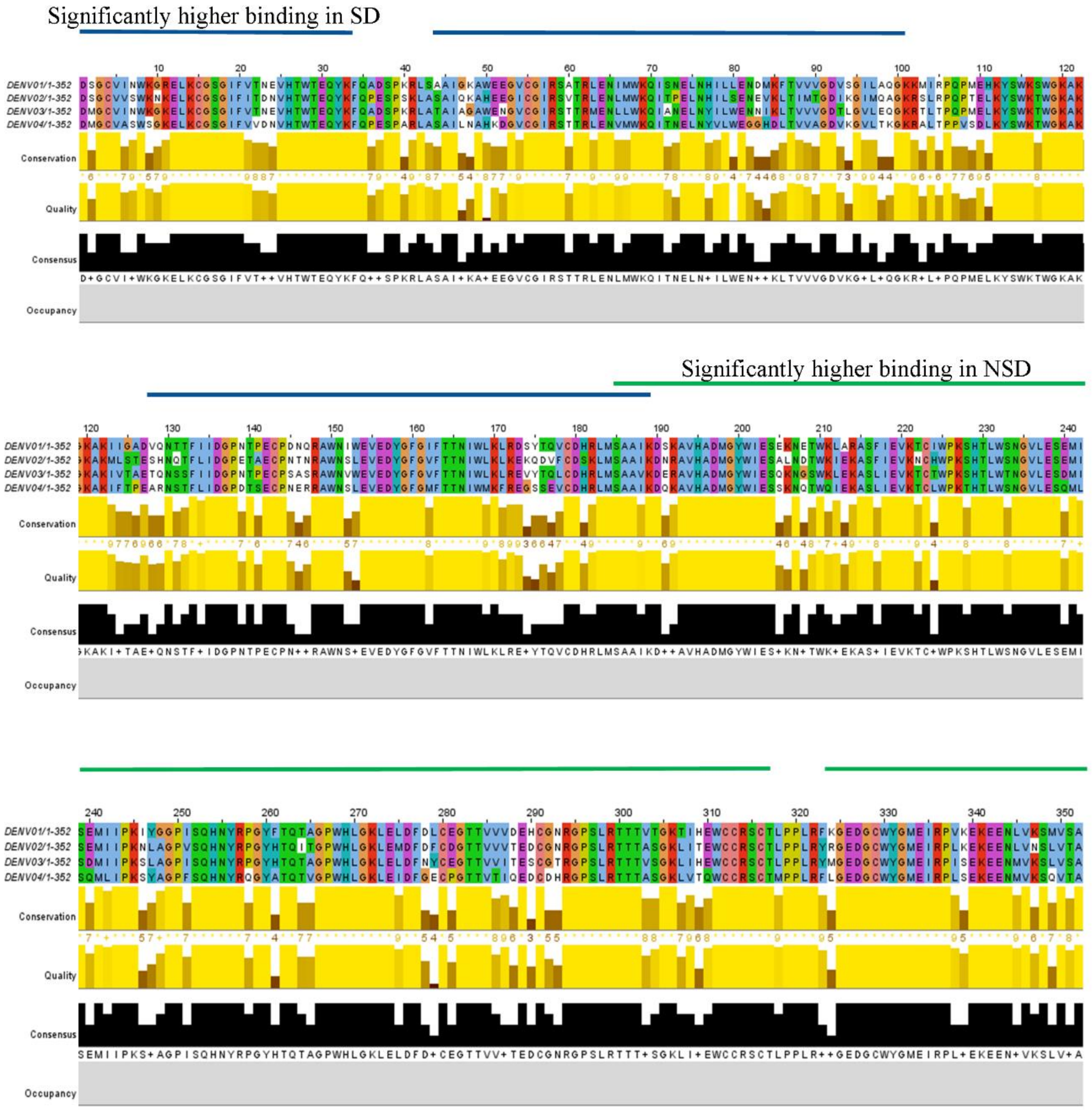

Supplementary figure 3: **Multiple sequence alignment of NS1 protein of four serotypes of DENV.** The blue bars represent NS1 regions where significantly higher binding was observed in individuals with past severe dengue infection (SD) and the green bars raepresent NS1 regions where significantly higher binding was observed in individuals with past non- severe dengue infection (NSD).

a

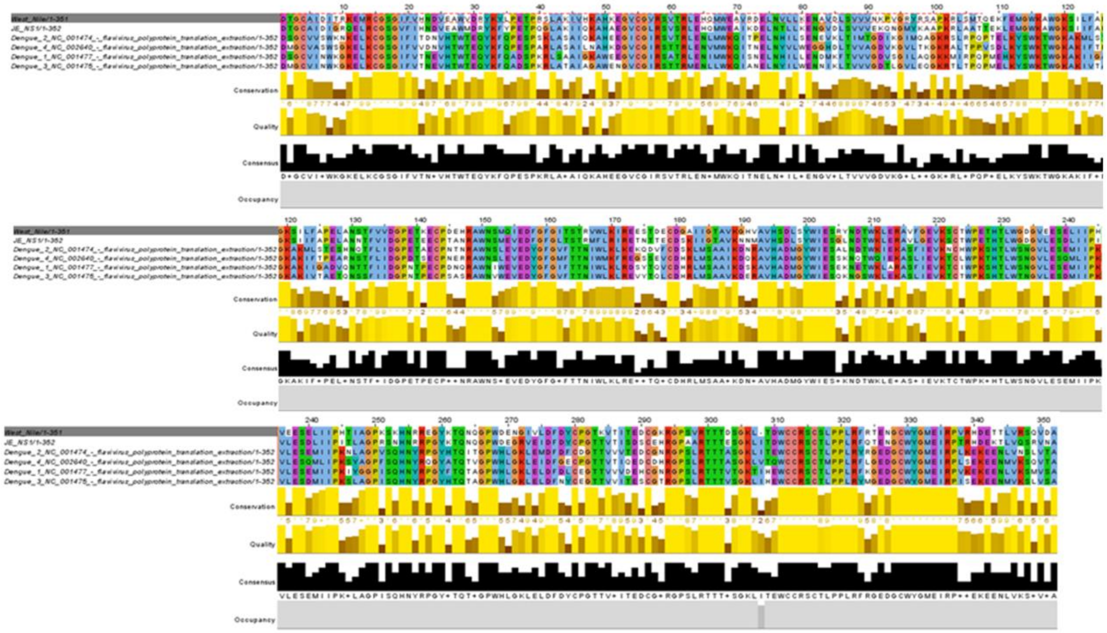
Conservation
Quality
Consensus
Occupancy

b

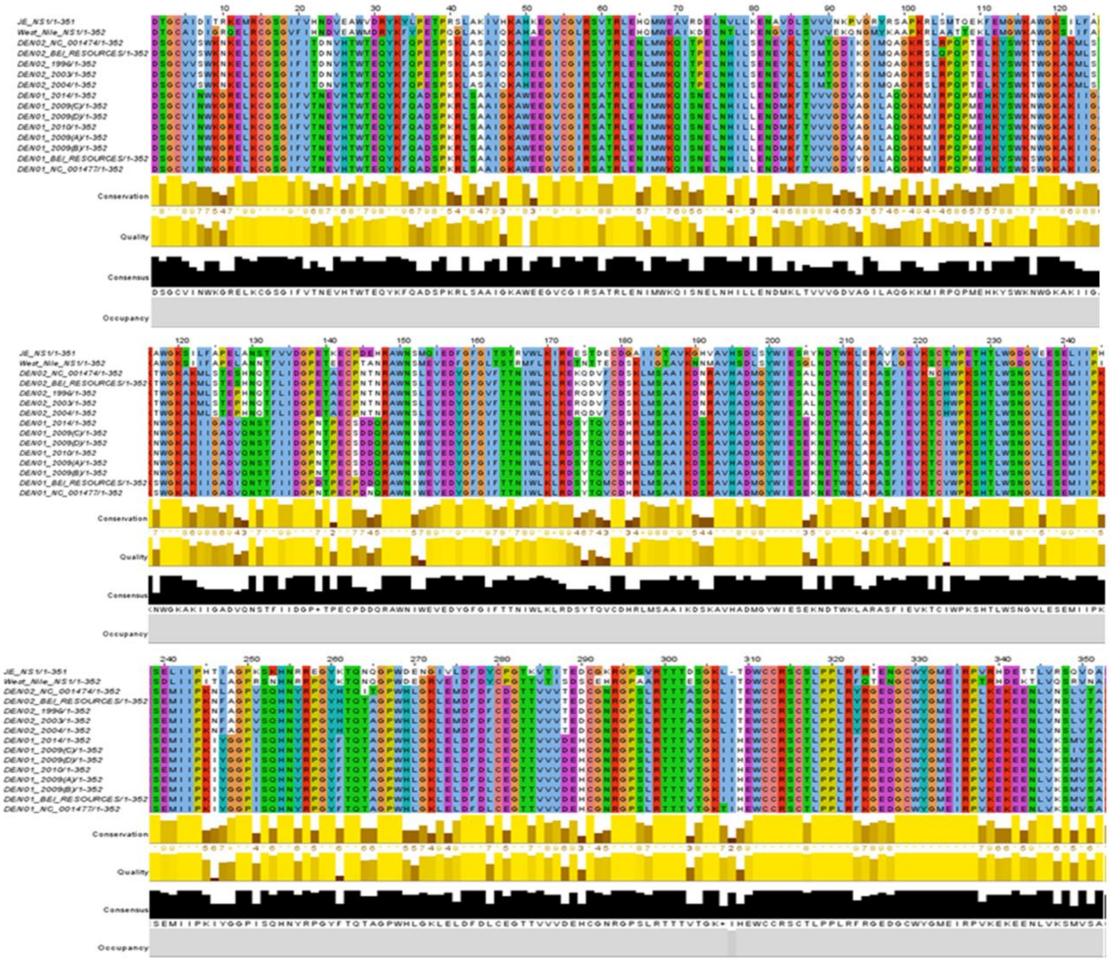
Conservation
Quality
Consensus
Occupancy

Supplementary figure 4: Multiple sequence alignment of NS1 protein of the DENVs, JEV and WNV. DENV1 to 4 NS1 sequences were aligned with JEV and WNV using Clustal Omega (a), DENV1 and DENV2 sequences identified in recent years were aligned with JEV and WNV (b)
